# The MyLO CRISPR-Cas9 toolkit: a markerless yeast localization and overexpression CRISPR-Cas9 toolkit

**DOI:** 10.1093/g3journal/jkac154

**Published:** 2022-06-16

**Authors:** Björn D M Bean, Malcolm Whiteway, Vincent J J Martin

**Affiliations:** Department of Biology, Centre for Applied Synthetic Biology, Concordia University, Montréal, QC H4B1R6, Canada; Department of Biology, Centre for Applied Synthetic Biology, Concordia University, Montréal, QC H4B1R6, Canada; Department of Biology, Centre for Applied Synthetic Biology, Concordia University, Montréal, QC H4B1R6, Canada

**Keywords:** MyLO, CRISPR, Cas9, yeast, toolkit, integration, microscopy, localization, colocalization, overexpression

## Abstract

The genetic tractability of the yeast *Saccharomyces cerevisiae* has made it a key model organism for basic research and a target for metabolic engineering. To streamline the introduction of tagged genes and compartmental markers with powerful Clustered Regularly Interspaced Short Palindromic Repeats (CRISPR) - CRISPR-associated protein 9 (Cas9)-based genome editing tools, we constructed a Markerless Yeast Localization and Overexpression (MyLO) CRISPR-Cas9 toolkit with 3 components: (1) a set of optimized *Streptococcus pyogenes* Cas9-guide RNA expression vectors with 5 selectable markers and the option to either preclone or cotransform the gRNAs; (2) vectors for the one-step construction of integration cassettes expressing an untagged or green fluorescent protein/red fluorescent protein/hemagglutinin-tagged gene of interest at one of 3 levels, supporting localization and overexpression studies; and (3) integration cassettes containing moderately expressed green fluorescent protein- or red fluorescent protein-tagged compartmental markers for colocalization experiments. These components allow rapid, high-efficiency genomic integrations and modifications with only transient selection for the Cas9 vector, resulting in markerless transformations. To demonstrate the ease of use, we applied our complete set of compartmental markers to colabel all target subcellular compartments with green fluorescent protein and red fluorescent protein. Thus, the MyLO toolkit packages CRISPR-Cas9 technology into a flexible, optimized bundle that allows the stable genomic integration of DNA with the ease of use approaching that of transforming plasmids.

## Introduction

The yeast *Saccharomyces cerevisiae* is a key model eukaryotic cell in part due to the ease of genetic manipulations resulting from its high rate of homologous recombination and the availability of stable plasmids. This led to the development of toolkits facilitating genetic manipulations, typically based on stable selectable markers (Sikorski and Hieter 1989; Longtine *et al.* 1998). However, the small set of available markers limited the number of manipulations possible without using methods to recycle markers based on counterselections (Alani *et al.* 1987; Storici and Resnick 2006) or the Cre-lox system (Jensen *et al.* 2014). The discovery of Clustered Regularly Interspaced Short Palindromic Repeats (CRISPR) and CRISPR-associated protein 9 (Cas9) has greatly improved the efficiency of genomic manipulations (DiCarlo *et al.* 2013; Doudna and Charpentier 2014). The Cas9 endonuclease can be directed to introduce a double-stranded break at a specific location determined by a guide RNA (gRNA). Subsequently, the cellular machinery attempts repair by either error-prone nonhomologous end joining (NHEJ) or homology-directed repair (HDR). The increased propensity for HDR makes genomic edits based on homologous recombination over 1,000 times more efficient, alleviating the need to integrate stable selectable markers (Ryan *et al.* 2014).

In yeast, catalytically active *Streptococcus* *pyogenes* Cas9 has now been applied in multiple toolkits and contexts. Combined Cas9-gRNA expression plasmids were made, and gRNA cloning methods were simplified (Ryan *et al.* 2014; Laughery *et al.* 2015; Levi and Arava 2020). Groups also focused on multiplexing CRISPR, allowing multiple simultaneous integrations with methods to introduce multiple guides and donors (Ryan *et al.* 2014; Horwitz *et al.* 2015; Jakočiūnas, Bonde, *et al.* 2015; Jakočiūnas, Rajkumar, *et al.* 2015; Mans *et al.* 2015; Zhang *et al.* 2019). Others updated a toolkit based on Cre-LoxP recycling of selectable markers with CRISPR to make a markerless system for introducing untagged genes for metabolic engineering (Ronda *et al.* 2015; Jessop‐Fabre *et al.* 2016). Further consolidation of guides and donor DNA has also enabled large-scale CRISPR-based screens (Bao *et al.* 2018; Guo *et al.* 2018; Sadhu *et al.* 2018).

While CRISPR-Cas9 systems have progressed, there have been limited efforts to establish a robust set of accompanying vectors for integrating compartmental markers and tagged or untagged genes of interest (GOIs) using Cas9. Thus, though CRISPR-Cas9 methodologies present advantages in terms of flexibility and efficiency, classic systems based on homologous recombination remain heavily utilized for protein tagging. Furthermore, plasmids are regularly used for the expression of fluorescent compartmental markers, leading to uneven expression within populations (Ryan *et al.* 2014).

Here, we present an optimized CRISPR-Cas9 toolkit for the rapid introduction of tagged genes and compartmental markers. First, we simplified the Cas9/gRNA expression vector developed by Ryan *et al.* to enable PCR-free Golden Gate cloning of gRNAs into vectors with 5 different selections (Ryan *et al.* 2014). We then established novel “split selection” markers that improve transformation efficiencies by allowing transformation of linearized pCAS vectors with intramolecular recombination generating a selectable marker. To complement the pCAS vectors, we developed a set of Golden Gate cloning-compatible plasmids that can be used to rapidly create integration cassettes that can introduce a GOI with 3 different N- or C-terminal tags at 3 expression levels. Importantly, these cassettes contain novel multipurpose homology arms that allow gRNA-based targeting to 7 different established safe harbor loci, helping ensure that an integration site is usually available. Finally, we generated a set of green fluorescent protein (GFP) or red fluorescent protein (RFP)-tagged compartmental markers to facilitate and standardize colocalization studies, aiming to impart the simplicity of plasmids to the stability of integration. Together, these optimized yeast pCas9 vectors and versatile integration cassettes form the Markerless Yeast Localization and Overexpression (MyLO) CRISPR-Cas9 toolkit.

## Materials and methods

### Plasmids and yeast strains

Most plasmids used were from the MyLO Toolkit collection constructed here. They are listed in Supplementary Table 1 and are available from Addgene with fully annotated maps included under the Resource Information section. Plasmids were made using Golden Gate cloning or homologous recombination in yeast as outlined in detail in Supplementary File 1 with primers listed in Supplementary Table 2. Yeast strains, listed in Supplementary Table 3, were made with CRISPR/Cas9-based transformations using MyLO Toolkit components. All plasmids were confirmed by sequencing and all integrants were confirmed by PCR.

### Fluorescence-based yeast transformation assay

Assays of transformation efficiency were performed by integrating a strong GFP expression cassette [pBBK93, *TDH3p-Neon(gfp)*] into wild-type yeast using a lithium acetate-based transformation protocol (Gietz and Schiestl 2007). Briefly, 3 optical density 600 nm (OD_600_) of log phase yeast were transformed by incubating for 30 min at 30°C and then 42°C in a 150 µL final volume with concentrations as in Gietz *et al.* Twenty percent of the reaction was plated and incubated 2 days at 30°C prior to imaging on a Safe Imager 2.0 (Invitrogen) blue light source to identify positive, green colonies. In some cases, colonies were counted using the Fiji (Schindelin *et al.* 2012) Color Threshold tool followed by the Analyze Particles tool.

### Fluorescence microscopy

Log-phase yeast grown in synthetic selective media were imaged on slides using a DMi6000B microscope (Leica Microsystems) with an HCX PL APO 63x oil objective, an Orca R2 CCD camera (Hamamatsu) and Volocity software (PerkinElmer). Images within each panel were evenly exposed and processed using FiJi (Schindelin *et al.* 2012) and Photoshop CC (Adobe) except for those in Fig. 5 where large fluctuations in intensity necessitated different exposure times.

### Western blot

Log-phase yeast were lysed by freezing a pellet, suspending them in Alternate Thorner Buffer (8M Urea, 5% SDS, 40 mM Tris 6.8, 0.1 mM EDTA, 0.4 mg/mL bromophenol blue, 1% β-mercaptoethanol) with glass beads, heating at 70°C for 5 min and then vortexing 1–2 min. An amount of lysate equivalent to 0.1 OD_600_ of yeast was run on an SDS-PAGE gel, transferred to nitrocellulose paper and blotted with mouse anti-hemagglutinin (HA; Abcam ab18181) followed by donkey anti-mouse conjugated to IR-Dye 800CW (Mandel Scientific 926-32212). The blot was imaged on an Odessey 9120 Infrared Imager (LI-COR).

### Flow cytometry

Log-phase yeast grown in synthetic complete media were measured on an Accuri C6 flow cytometer (BD Biosciences). For each sample, 10,000 events were collected, and the mean green fluorescence values of the complete ungated populations were recorded.

## Results

### Optimizing use of Cas9 expression vectors in *S. cerevisiae*

To create a set of flexible Cas9-gRNA expression cassettes, we modified an established pCAS vector (Addgene #60847; Ryan *et al.* 2014). This vector expresses *S. pyogenes* Cas9 and a gRNA comprising of the 5′-cleaving hepatitis delta virus ribozyme, a 20 bp protospacer responsible for Cas9 targeting and a scaffold mediating Cas9 interactions. *Streptococcus* *pyogenes* Cas9 targets regions that match the protospacer sequence and are followed by the protospacer adjacent motif (PAM) -NGG-, cutting 3 bp upstream of the PAM (Gasiunas *et al.* 2012; Jinek *et al.* 2012). We first substituted the *Escherichia* *coli-*yeast Kanamycin resistance (KanR) cassette for a Hygromycin resistance (HygR) cassette and, in each plasmid, replaced the protospacer with a cleanly excisable stuffer containing an *Not*I and 2 *Bsa*I cut sites, respectively, generating pBBK94 and pBBK95.

**Fig. 1.**
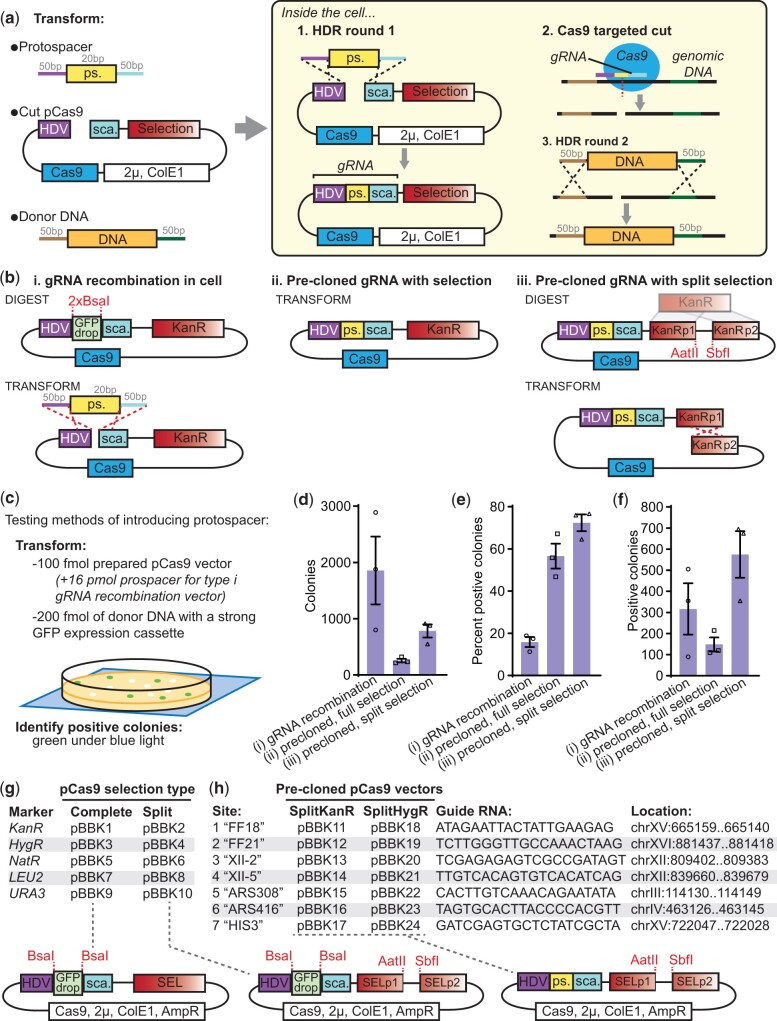
New Cas9 expression vectors for improved markerless CRISPR transformations. a) Classic approach for integrating DNA in *S. cerevisiae* with CRISPR-Cas9. Three DNA fragments are cotransformed: a protospacer (ps.), a cut Cas9 vector and donor DNA. In the cell, (1) the pCAS vector is repaired, introducing the protospacer upstream of a scaffold (sca.), together creating a complete gRNA. (2) The gRNA directs Cas9 to cut at a site specified by the protospacer, which is then [3] repaired by the cell using the donor DNA. b) Alternative strategies for introducing the gRNA and Cas9 beyond (i) the classic recombination approach include either precloning the protospacer into a Cas9 vector with a (ii) complete selection or (iii) a “split selection” that must be repaired in the cell prior to expression. c) To test the efficiency of the 3 approaches, 100 fmol (569–600 ng) of each Cas9 vector, with 16 pmol (1,248 ng) protospacer for the type i gRNA recombination vector, and 200 fmol (730 ng) of a strong GFP expression integration cassette (*TDH3p-GFP*) were transformed. Green colonies under a blue light indicated successful transformations. The resultant (d) total number of colonies, (e) percent of colonies that were positive and (f) total number of positive colonies were determined. *n* = 3; data presented as mean ± SEM. g) A series of Cas9 vectors with 5 types of complete or split selections. h) A series of split selection Cas9 vectors with precloned gRNAs for 7 sites used in this toolkit.

These vectors can be used by excising the stuffer and cotransforming the pCAS with donor DNA and a protospacer fragment made by dimerizing 2 primers (Fig. 1a). Inside the cell HDR introduces the protospacer into the pCAS allowing expression of Cas9 and a complete gRNA. Selecting for pCAS is sufficient for identifying integrants, and once identified, the selection can be dropped allowing the cells to discard the plasmid resulting in a “markerless” genome edit. While flexible, this approach depends on error-free recombination to generate the gRNA. Unfortunately, either NHEJ occurring at the guide site or errors in gRNA primers can result in failure to activate Cas9 and the production of nonmodified colonies.

Consequently, we investigated improving transformation efficiency by modifying how the protospacer is introduced. Initial tests of precloned protospacers resulted in substantially lower transformation efficiencies, leading us to hypothesize that linearization of the pCAS is required for high efficiencies (data not shown). Therefore, we shifted the site of linearization to the selection cassette such that selection would depend on repair. We built a novel KanR-based “split selection” (SplitKanR) cassette by fusing the first two-thirds of KanR to the final two-thirds with a short linker containing restriction enzyme cut sites (Fig. 1b-iii). The original pCAS KanR marker was replaced with SplitKanR, and an ampicillin resistance cassette for *E. coli* selection was added. Transformations with this plasmid linearized at the SplitKanR should result in increased efficiency because there is more homology for HDR-based repair, the homology is intramolecular and the process selects for error-free repair.

We compared the transformation efficiencies of the 3 gRNA introduction strategies: recombination at the gRNA, precloning the gRNA with a complete selection, and precloning the gRNA with a split selection (Fig. 1, b and c). To do so, we transformed a strong GFP expression cassette into the safe harbor site FF18 and identified positive colonies by their green fluorescence (Fig. 1h). Transformation tests used 200 fmol of donor DNA (from pBBK93), 100 fmol of prepared pCas9 vector and, for the first strategy, a 160x molar excess of the protospacer. The gRNA recombination vector resulted in the most colonies, while the precloned full and split selection vectors, respectively, yielded 14% and 42% as many colonies (Fig. 1d). This is consistent with reports that linearization improves CRISPR-Cas9 transformations and suggests that either the protospacer excess or NHEJ-based repair at the protospacer site further increases the number of transformants (Horwitz *et al.* 2015; Guo *et al.* 2018). However, the protospacer recombination approach yielded only 16% positive colonies (green under blue light), roughly one quarter that of either precloned approach (Fig. 1e). The colonies that were negative with the precloned approaches were likely a result of genomic repair by NHEJ. Thus, the precloned split selection pCAS yielded the most positive colonies, highlighting that this novel approach can take advantage of linearization-mediated transformation efficiency improvements while avoiding the generation of inactive gRNAs (Fig. 1f).

We next generated and validated a series of pCAS vectors with a variety of complete and split selections for either single-step gRNA recombination transformations or precloning of gRNAs (Fig. 1g). We included vectors for the gRNA recombination method as it is faster and easier for one-off transformations or efficient guides. To facilitate gRNA cloning, all vectors contain a *Bsa*I-flanked GFP dropout cassette at the protospacer site for PCR-free Golden Gate cloning (see Supplementary File 2 for cloning guidelines). The *URA3* selectable marker in pBBK9 is also *BsmB*I-flanked to simplify introduction of alternate selections with Golden Gate cloning. We also cloned 7 guides into both the SplitKanR and SplitHygR vectors (Fig. 1h). These guides correspond to 6 commonly used safe harbor sites FF18/21 (Flagfeldt *et al.* 2009), XII-2/5 (Jessop‐Fabre *et al.* 2016), ARS308/416 (Reider Apel *et al.* 2017), and one that targets *HIS3* enabling screening. Collectively, our Cas9 vectors offer improved transformation efficiencies with flexibility in strategy and selections.

### A toolkit for rapid construction of integration cassettes

Classic approaches for expressing integrated tagged proteins often focus on introducing tags at the native locus of the GOI (Fraczek *et al.* 2018). While this can be done markerlessly with CRISPR-Cas9, it requires the case-specific identification of a Cas9 cut site proximal to the 5′ or 3′UTR of the GOI and the amplification of donor DNA containing the tag with flanking homology specific to that site. Unfortunately, these cut sites can have lower efficiencies, and as the distance of the cut site from the gene increases, the probability of the desired recombination event decreases (Supplementary File 2). Furthermore, expression of heterologous genes requires an alternate approach, and colocalization studies often favor the ease of plasmid-based expression even though copy number variations cause significant differences in intensity between cells (Ryan *et al.* 2014). CRISPR-Cas9 offers the alternative of easily introducing a complete expression cassette, containing the tagged GOI, at other loci such as the nondisruptive safe harbor sites (Fig. 1h).

**Fig. 2.**
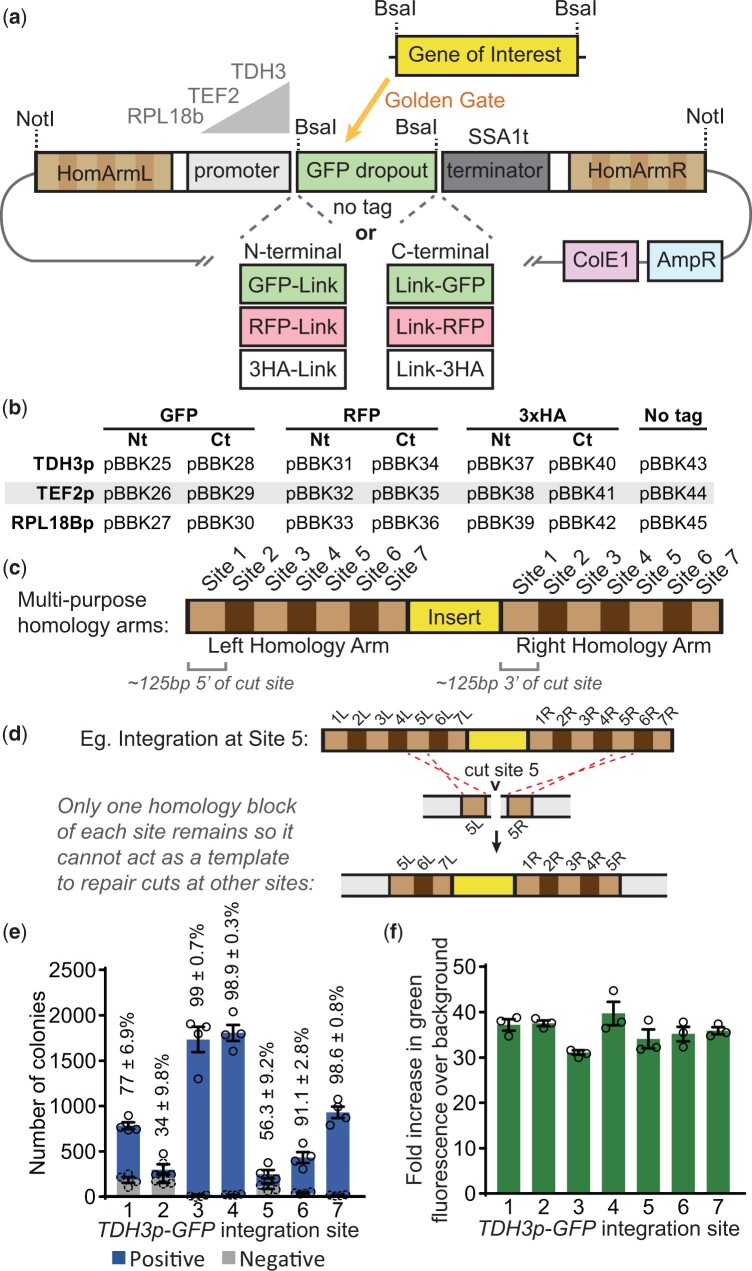
A set of markerless integration cassettes for introducing untagged, GFP-, RFP-, or 3HA-tagged GOIs with multipurpose targeting sequences. a) Schematic of integration cassettes with left and right homology arms flanking a promoter at one of 3 strengths, a GFP dropout and a terminator. Some include N- or C-terminal GFP, RFP, or 3HA tags as indicated. Golden Gate cloning with a BsaI-flanked gene of interest allows introduction of targets. b) The integration cassettes parent vectors included in this kit. c) The homology arms consist of roughly 125-bp regions either upstream (left arm) or downstream (right arm) of each Cas9 cut site described in Fig. 1h, arranged in numerical order. d) An example integration at Site 5 indicating that afterwards only 1 homology block of the other sites remains so this integrant will not interfere with subsequent transformations into those sites. e) Transformation efficiencies at the different sites were determined by integrating a *TDH3p-GFP* cassette at each site and counting the total number of colonies as well as the number of colonies that were green under a blue light (positive). Transformations used 100 fmol (600–690 ng) of a precloned split selection pCAS from Fig. 1h and 200 fmol (730 ng) of the expression cassette. *n *=* *4, Twice each with SplitKanR and SplitHygR vectors. f) To assess expression levels at the integration sites, *TDH3p-GFP* was introduced at each site, and the increase of green fluorescence over the wild-type background was measured by flow cytometry. *n *=* *3; 10,000 cells/strain/replicate. Data presented as mean ± SEM.

To facilitate this approach, we adopted a modular cloning scheme to generate plasmids that simplify building expression cassettes for untagged, GFP-, RFP-, or HA-tagged GOIs (Lee *et al.* 2015; Fig. 2a). Each parent plasmid contains homology arms, a promoter, optionally a tag, a terminator, and a *Bsa*I-flanked GFP dropout for introducing a GOI by Golden Gate cloning. All tags contain glycine- and serine-rich linkers (Supplementary File 1). Once assembled, *Not*I digestion linearizes complete integration cassettes for transformations. Parent plasmids are available featuring moderate to strong promoters (*RPL18Bp* < *TEF2p* < *TDH3p*) and with optional N- or C-terminal tags (Fig. 2b). Both the GFP and the RFP are recently developed bright variants, ymNeonGreen and ymScarletI, respectively, (Botman *et al.* 2019). Microscopy and flow cytometry were used to verify ymNeonGreen was brighter than alternative GFP variants Envy and ZsGreen1 (Supplementary Fig. 1, a and b).

These integration cassette parents feature unique multipurpose homology arms (Fig. 2c). Each arm contains a series of roughly 125 bp sections homologous to regions upstream (left arm) and downstream (right arm) of the 7 commonly used integration sites corresponding to the precloned gRNAs in this kit (Figs. 1g and 2c). The sequential orientation of Sites 1–7 on each arm means that once a cassette is integrated at one site it cannot act as a repair template for subsequent cuts at the other 6 sites (Fig. 2d). Therefore, existing integrated cassettes cannot interfere with subsequent integrations at other sites. This allows up to 7 integration cassettes with the same homology arms to be sequentially introduced into a strain, facilitating gene dosage and colocalization experiments.

To validate this system, the KanR and HygR precloned split selection pCAS vectors (Fig. 1g) were used to integrate a strong GFP expression cassette (as in Fig. 1c) into each integration site (Fig. 2e). Transformations resulted in 250–1,800 colonies, with integrations into Sites 3, 4, and 7 resulting in greater than 98% positive colonies. Integrations into Sites 2 and 5 yielded fewer than 60% positive colonies, which was unexpectedly poor relative to previous reports (Reider Apel *et al.* 2017; Bourgeois *et al.* 2018), though still sufficient for straightforward integration of cassettes. The drop in efficiency may reflect some structural feature of the homology arms or GFP expression cassette. The latter is likely for Site 2 as subsequent transformations into Site 2 for Fig. 4c yielded 88% positive colonies by PCR (data not shown). Having confirmed the cassette could be integrated at all sites, expression levels between sites were compared. Expression levels, assessed by measuring green fluorescence by flow cytometry, were similar between sites at an average of 36 times the background fluorescence (Fig. 2f, Supplementary Fig. 1c). Together, the cassettes developed here can be integrated at 7 gRNA-determined sites and result in expression levels uninfluenced by the integration site.

**Fig. 3.**
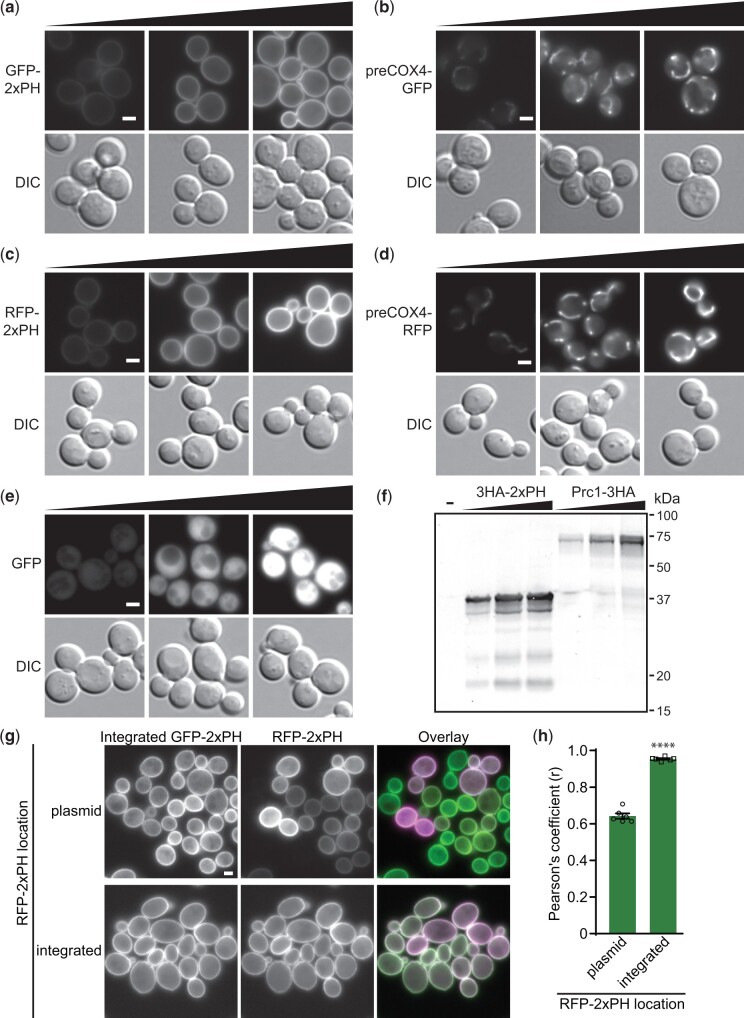
Validation of integration cassettes. All parent integration cassettes presented in Fig. 2b were tested using compartmental markers. GFP-tagging cassettes were used to introduce (a) N-terminally tagged plasma membrane marker 2xPH or (b) C-terminally tagged mitochondrial preCox4 into Site 1 and the resultant strains were imaged. Black triangles in all panels indicate increasing promoter strengths; left to right: *RPL18Bp*, *TEF2p*, *TDH3p*. *n *=* *1. c) RFP-tagged 2xPH and (d) preCox4 were also introduced at Site 1 and the strains were imaged. *n *=* *1. e) GFP was introduced into untagged cassettes that were integrated at Site 1 prior to imaging. *n *=* *2. f) HA-tagging cassettes were validated by introducing N-terminally 3HA-tagged 2xPH (37 kDa) or C-terminally 3HA-tagged vacuolar marker Prc1 (64 kDa) into Site 1 followed by preparing cell lysates, resolving them by 12% SDS-PAGE and immunoblotting with anti-HA. “-” indicates a wild-type control. *n *=* *1. g) Uniformity of expression was determined by comparing RFP-2xPH expressed from a low-copy yeast centromere plasmid or after genomic integration in a strain with integrated GFP-2xPH. h) Colocalization, assessed by Pearson’s coefficients, between GFP- and RFP-2xPH in the strains improved significantly when both markers were integrated. Two-tailed unequal variance *t*-test. *n* = 6, >153 cells/condition/replicate. ****P < 0.0001; data presented as mean ± SEM. Scale bars represent 2 µm.

To confirm the functionality of all parent cassettes in Fig. 2b, compartmental markers were cloned into each and then transformed into Site 1. For N-terminal tagging with fluorophores, a plasma membrane marker comprised of 2 phospholipase Cδ Pleckstrin homology domains (2xPH; Levine and Munro 2002) was used while the mitochondrial marker preCOX4 (Sesaki and Jensen 1999) was used for C-terminal tagging. When integrated, microscopy showed GFP-tagged (Fig. 3, a and b) and RFP-tagged (Fig. 3, c and d) versions were expressed with the expected cellular distributions. The functionality of tag-free cassettes was demonstrated by introducing GFP (Fig. 3e). HA tagging cassettes were tested by introducing 2xPH and the vacuolar-localized protein Prc1 (Huh *et al.* 2003) and Western blotting (Fig. 3f). In all cases, expression levels predictably corresponded to the promoter used, demonstrating the integration cassettes can be used effectively to integrate target GOIs at adjustable expression levels.

To assess the evenness of expression from our integrated cassettes, we introduced RFP-2xPH into our GFP-2xPH strain either on a low-copy plasmid or into the genome at Site 2 (Fig. 3g). As expected, there was substantially more variation in RFP-2xPH expression from the plasmid with some cells varying in fluorescence intensity or not expressing any detectable RFP-2xPH. This difference was quantifiable as a significant drop in the Pearson’s coefficient between the red and green channels when RFP-2xPH is expressed from a plasmid (Fig. 3h).

### Integration cassettes with GFP- or RFP-tagged localization markers for colocalization studies

**Fig. 4.**
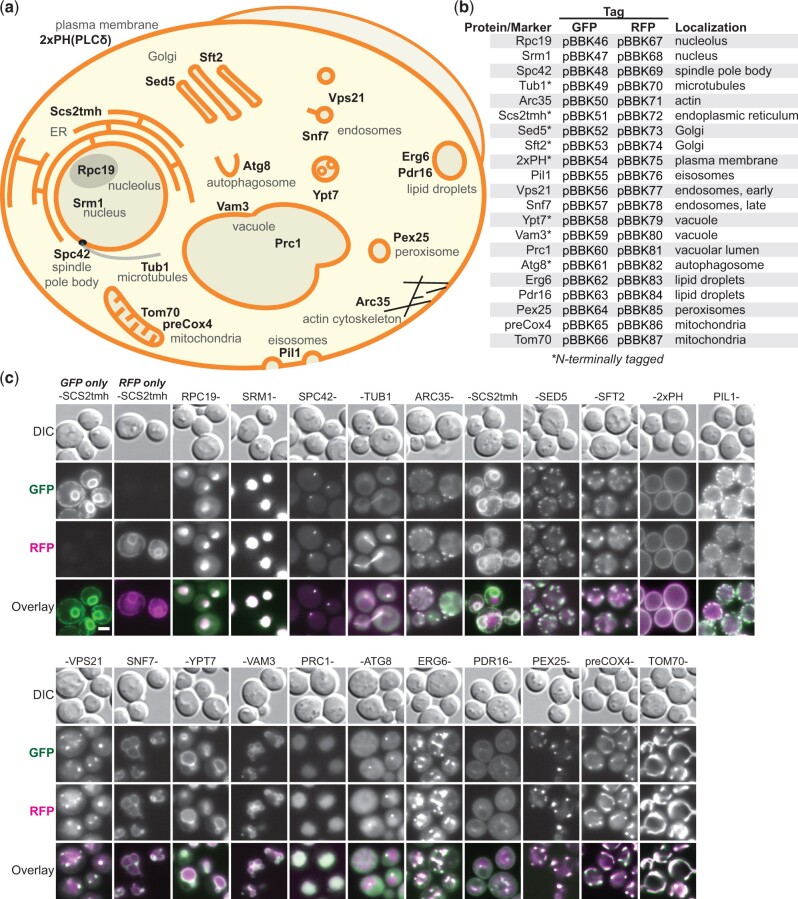
A library of integration-ready GFP- or RFP-tagged compartmental markers. a) Schematic of a yeast cell with distinct structures and their associated markers indicated. Markers are complete yeast proteins except the transmembrane helix of Scs2 (Scs2tmh), a fusion of 2 phospholipase Cδ_1_ pleckstrin homology domains [2xPH(PLCδ)] and the Cox4 target peptide (preCox4). b) Constructed integration cassettes expressing the indicated GFP- or RFP-tagged markers from *RPL18Bp* promoters. c) The integration cassettes were introduced at Site 1 (GFP) and Site 2 (RFP) and colocalization was monitored by fluorescence microscopy. *n *=* *2. Scale bar represents 2 µm.

While fluorescently tagging a protein can reveal positional information, accurate identification of localization typically requires colocalization with a compartmental marker. To facilitate this type of work, we created a library of integration cassettes containing GFP- or RFP-tagged markers for 15 subcellular locations expressed from the *RPL18B* promoter (Fig. 4, a and b). The 21 markers selected were primarily well-characterized full-length native proteins with localization confirmed in large-scale screens (Huh *et al.* 2003; Chong *et al.* 2015). Additionally, the transmembrane helix of Scs2 (Scs2tmh) was used to mark the ER (Loewen *et al.* 2007) and the 2xPH (Levine and Munro 2002) and preCox4 (Sesaki and Jensen 1999) markers were used for the plasma membrane and mitochondria, respectively. Markers were C-terminally tagged except in cases where that had been shown to be disruptive, in which case they were N-terminally tagged (Chong *et al.* 2015; Fig. 4b, asterisks).

To validate the compartmental markers, we imaged strains with GFP- and RFP-tagged versions of each marker. The GFP and RFP cassettes were integrated at Sites 1 and 2, respectively, and colocalization was assessed by fluorescence microscopy (Fig. 4c). We observed both even expression levels and strong colocalization between GFP and RFP versions of each marker. Controls with only GFP-Scs2tmh or RFP-Scs2tmh demonstrated colocalization was not an artifact of bleed-through between channels. While there was some variation in marker intensities, consistent with variable protein stabilities, intensity was consistent enough to image all strains with the same exposure times (400 ms GFP, 800 ms RFP), streamlining microscopy. In some cases, the relative intensities of the markers varied with either the GFP (Pil1) or RFP (Tub1, Arc35) markers being brighter, suggesting that in some cases one of the tags is more destabilizing or disruptive. Together, these markers demonstrate the ease of integrating tagged genes with the MyLO toolkit and should serve as useful tools for future colocalization work.

### Applying the toolkit for overexpression

**Fig. 5.**
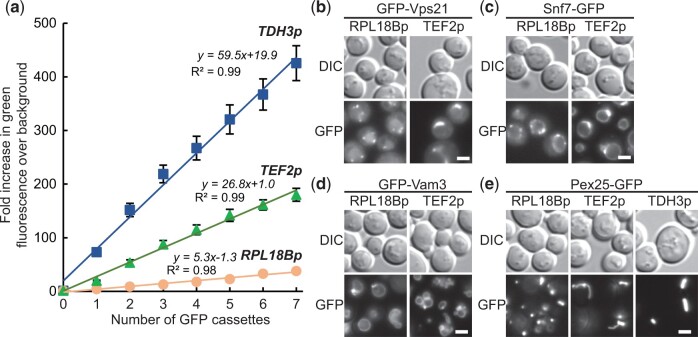
Using integration cassettes for overexpression. a) Stepwise increases in GFP expression from cassettes with *RPL18Bp*, *TEF2p*, and *TDH3p* promoters sequentially introduced at Sites 1 through 7 was monitored by measuring the increase of green fluorescence over the wild-type background with a flow cytometer. *n *=* *3, 10,000 cells/strain/replicate. Fluorescence microscopy of yeast with GFP-tagged markers (b) GFP-Vps1, (c) Snf7-GFP (d) GFP-Vam3, and (e) Pex25-GFP displaying changes in localization as promoter strength increases *RPL18Bp* < *TEF2p* < *TDH3p*. Exposure times were adjusted to compensate for differing expression levels and highlight localizations. *n *=* *2. Scale bars indicate 2 µm. Data presented as mean ± SEM.

The toolkit facilitates overexpression experiments by allowing sequential integration of a single cassette. Given the even expression across integration sites (Fig. 2f), the relationship between cassette number and expression level should be linear if the cellular machinery mediating expression is not limiting. To establish this relationship, we sequentially integrated 7 copies of GFP expression cassettes driven by *RPL18B*, *TEF2*, and *TDH3* promoters at sites one through 7. Measuring the fluorescence of all strains, including intermediates, confirmed a linear relationship with copy number for each of the promoters used (Fig. 5a). The strains with *TDH3p* and *TEF2p* cassettes were downward deflected and fit better with negative second-order polynomials, indicative of expression limitation due to factors such as transcription factor titration or stress on protein biosynthetic machinery. This experiment also showed *TEF2p* is 5x and *TDH3p* is 11x stronger than *RPL18B*.

While multiple copies can be useful for gene dosage experiments or metabolic engineering, localization can be disrupted by expressing a gene with a stronger promoter. We observed such overexpression-induced mislocalization with some compartmental markers, as all were initially *TEF2p*-expressed to reduce exposure times. For example, the early endosomal Rab5 GTPase GFP-Vps21 shifted from small puncta to larger peri-vacuolar bars when *TEF2p*-expressed (Fig. 5b). Likewise, *TEF2p*-expressed Snf7-GFP, a component of the endosomal sorting complexes required for transport (ESCRT-III) complex, accumulated in larger vacuolar puncta and on the vacuolar rim (Fig. 5c). In other cases, overexpression disrupted compartmental shape. *TEF2p*-expression of the target soluble N-ethylmaleimide-sensitive factor attachment protein receptor (tSNARE) GFP-Vam3 led to modest vacuolar fragmentation (Fig. 5d). Strikingly, *TEF2p* expression of the peroxin Pex25-GFP resulted in a shift to bars with elongated tubules that sometimes extended from mother to daughter cells (Fig. 5e). Further increasing expression by switching to the *TDH3* promoter resulted in loss of the elongated tubules and formation of intense bars. In all cases equivalent RFP-tagged markers localized similarly (Supplementary Fig. 1, d–g). In contrast, other tagged proteins maintained wild-type localization when overexpressed, a subset of which we include as bright markers for the plasma membrane, endoplasmic reticulum, and cytosol (pBBK88-93). These experiments demonstrate that overexpression studies can be conducted using the included promoters and homology arms.

## Discussion

**Fig. 6.**
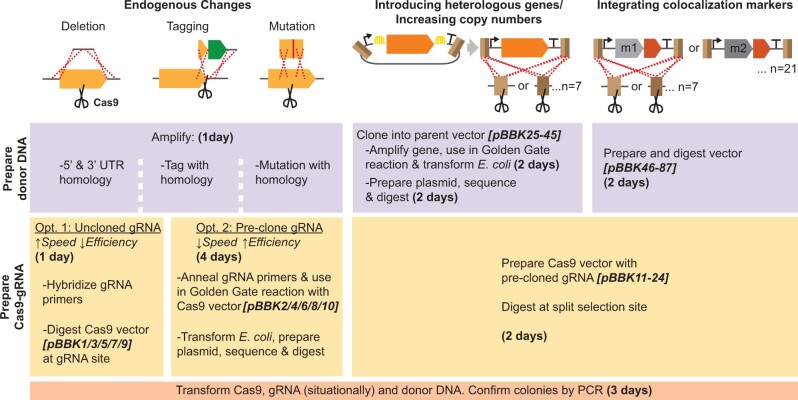
Overview of MyLO toolkit use cases and workflow. The toolkit facilitates both endogenous changes as introduction of heterologous genes. In all cases, donor DNA and Cas9 vectors require preparation (upper and middle bars, respectively), which can be done concurrently. Time required and vectors available are dependent on the method selected as indicated. Where no cloning step is required, components can be prepared and transformed in a single day. Dashed line, crossover event; scissors, Cas9 cut; Arch, Golden Gate reaction; m, compartmental marker.

This work provides an expansive toolkit optimized for Cas9-mediated introduction of tagged genes and compartmental markers into the yeast genome. Our pCAS-gRNA vectors provide flexibility with 5 selectable markers and the option of using common precloned gRNAs or introducing new guides by either Golden Gate cloning or homologous recombination in yeast. Furthermore, we introduce novel split selection markers, demonstrating that linearizing pCAS vectors prior to transformation and directly selecting for their recombination boosts transformation efficiency. These pCAS vectors are complemented by a series of integration cassette parent vectors, simplifying the cloning of untagged and GFP-, RFP-, or HA-tagged genes into expression cassettes, each of which can be integrated at any of 7 sites. Also included is a collection of integration vectors with GFP- or RFP-tagged compartmental markers for use in colocalization studies. The MyLO toolkit streamlines markerless genomic manipulations (Fig. 6), seeking to approximate the ease of introducing a plasmid without the associated drawbacks, to support the rapid construction of complex strains for basic and applied yeast research.

There is substantial variation in how the core requirements for Cas9-based transformations, introducing Cas9 with variable gRNAs and donor DNA, are achieved. Some approaches combine all elements on single plasmids (Bao *et al.* 2015; Vyas *et al.* 2018) whereas others rely on preloading a strain with either the Cas9 alone (DiCarlo *et al.* 2013; Jessop‐Fabre *et al.* 2016) or, with the use of an inducible Cas9 cassette, together with a gRNA (Degreif *et al.* 2018). While both approaches work well, they are either cloning intensive or require longer transformation protocols. In contrast, cotransforming a combined pCas9-gRNA vector with a donor, as we and others (Laughery *et al.* 2015; Generoso *et al.* 2016) have pursued, requires only a simple protospacer cloning step before a one-step yeast transformation. Given the utility of this approach, we developed a set of these vectors with a variety of markers, though we note that Addgene contains extensions to the Laughery *et al.* system and unpublished vectors from the Ellis lab (pWS158, 171-176) that fill similar niches. The availability of multiple markers is important for marker cycling, where dropping a selection after a transformation allows a plasmid to be lost fast enough that every third, or in some cases second, transformation can be done with the same selectable marker on the pCAS without needing a curing step. Lastly, we simplified this pCas9-gRNA strategy by making protospacer cloning optional, precloning common guides, and ensuring all cloning is Golden Gate compatible.

We found that linearization of the Cas9 vectors improved transformations (Fig. 1). This, together with previous reports of gap repair increasing the percent of positive colonies (Horwitz *et al.* 2015), or both the number of colonies and the percent positive (Guo *et al.* 2018), could be explained by 2 mechanisms. First, as previously suggested, requiring gap repair of the Cas9 vector ensures that the cell is competent for homologous recombination, a prerequisite for efficient introduction of the donor DNA at the target site (Horwitz *et al.* 2015). Consistent with the expectation of a higher frequency of positive colonies, we observed a 28% increase in the percent of positive colonies with our split selection pCas9, relative to the circular vector. Second, the topology of linear DNA can promote improved transformation efficiencies, likely through increased DNA uptake (Raymond *et al.* 1999). Both this work, where linearized vectors resulted in 4–7x more colonies than transformations with circular pCas9, and that of Guo *et al.* support this mechanism as an important factor in improving Cas9-based transformation efficiency. Though our system uniquely implements an intramolecular gap repair strategy, the extent to which this boosts transformation efficiencies relative to intermolecular repair at either the marker (Horwitz *et al.* 2015; Guo *et al.* 2018) or to nonfunctional regions flanking the gRNA expression site (unpublished Ellis lab system; see pWS158 on Addgene) remains to be determined. Irrespective of transformation efficiency improvements, intramolecular gap repair simplifies usage as only a single digested pCas9-gRNA vector is needed for our approach, as opposed to requiring gRNA introduction on separate DNA. Collectively, there is now strong evidence that gap repair improves CRISPR-Cas9 methods.

Our extended set of integration cassettes, ready for introducing a tagged or untagged GOI, provides a compromise between flexibility and simplicity. The use of a Golden Gate cloning strategy compatible with the modular cloning toolkit (Lee *et al.* 2015) should simplify the introduction of genes from existing vectors (see Supplementary File 2 for cloning protocols). Furthermore, it should promote interchangeability with a recent extension of the modular cloning toolkit capable of multiplexing and designed for more variable markerless CRISPR-based applications (Otto *et al.* 2021). In contrast to these alternative systems, the MyLO kit sacrifices some versatility by providing prebuilt integration cassettes ready to introduce a GOI in a limited design space, 3 promoters with and without tags. This greatly simplifies cloning and minimizes sequencing requirements. Additionally, by avoiding multiplexing, we were able to introduce the unique multicomponent homology arms that allow gRNA-guided targeting of a single cassette to any of 7 sites. This allows many combinations of MyLO integration cassettes to be introduced both sequentially and repeatedly, without concern for clashing homology arms or the unavailability of a specific site, a frequent concern in metabolic engineering. Though crossover events could hypothetically occur between our integrated cassettes, in all cases where colonies were assayed by PCR, we observed the expected phenotype, including after 7 rounds of sequential transformations, suggesting the cassettes have high fidelity and stability.

The stability of our integrants is in line with previous studies on the stability of genomic DNA. Many metabolic engineering groups have previously observed stable expression of genes introduced into the established safe harbor sites we target, and Jessop‐Fabre *et al.* observed that after 5 generations all isolates tested maintained a genomically integrated GFP expression cassette (Flagfeldt *et al.* 2009; Jessop‐Fabre *et al.* 2016; Reider Apel *et al.* 2017). Multiple instances of our integration cassettes will share 5′ and 3′ homology which might impact stability. However, based on the stability and tolerance of the >377 kb of retrotransposon-related insertions already present in the *S. cerevisiae* genome, which frequently contain homology in the form of long terminal repeats, the effect of homology between instances of our cassettes should be minimal (Kim *et al*. 1998). Work by Koszul *et al.* on a yeast strain with an 115 kb internally translocated duplication further established the stability of genomic DNA (Koszul *et al*. 2006). They replaced a single copy of a gene within the duplication with *URA3* and then looked for colonies on plates with the *URA3* counterselection 5-floroorotic acid, which would have lost *URA3* function. They found that even with tens of kilobases of homology, a conversion event is only 2- to 3-times more frequent than an inactivating *URA3* point mutation. The ability of our toolkit to provide easy access to the stability of genomic integration represents a significant advantage over using unstable plasmids (Fig. 3, g and h).

We used the integration cassette parents to make an extended set of GFP- and RFP-tagged compartmental markers designed to provide the even level of expression of integrations while approximating the ease of a plasmid transformation. Indeed, we found that expression levels were exceptionally uniform between cells with a given marker, though intensity did vary between markers expressed from the same promoter. This variation can be explained by other factors that affect protein abundance including localization and interactions, codon usage, and the presence of degradation signals (Martin-Perez and Villén 2017; Tuller *et al*. 2007; Varshavsky 2019). Given the comparable expression levels, it should be possible to build tester strains with multiple morphologically distinct compartments, such as the ER, endosomes, and the plasma membrane, simultaneously tagged to rapidly identify the localization of a new protein. Our localization system does have the standard caveats associated with introducing second copies of genes: essential proteins inactivated by tagging will be possible to visualize though they may be mislocalized, and the untagged version of some proteins may partially outcompete the tagged version for recruitment to a given compartment. The ability to rapidly introduce a uniformly expressed compartmental marker for colocalization should greatly facilitate localization studies and automated image analysis.

Initial overexpression of our compartmental markers inadvertently yielded insights into the function of several proteins (Fig. 5, b–e). Excess GFP-Vps21 accumulated in puncta near the vacuole, and given that this Rab5 GTPase must be activated for membrane recruitment, our observation suggests a bias toward Rab5 activation (Lachmann *et al.* 2012; Nickerson *et al.* 2012; Cabrera and Ungermann 2013). While Snf7-GFP accumulated at perivacuolar puncta consistent with previous reports (Raymond *et al.* 1992; Froissard *et al.* 2007; Bean *et al.* 2015), we also observed Snf7-GFP on the vacuolar membrane, supporting a recent report of ESCRT function at the vacuole (Yang *et al.* 2021). Overexpression of Vam3-GFP, a tSNARE that mediates vacuolar fusion (Srivastava and Jones 1998; Lürick *et al.* 2015), resulted in vacuolar fragmentation, suggesting that the ratio of the SNARE to other fusion components is important for function (Alpadi *et al.* 2013; Lürick *et al.* 2015). Lastly, increasing Pex25-GFP levels led to enlarged peroxisomes with elongated tubules and then to intense bar-shaped structures. These phenotypes are likely linked to the role of Pex25 in peroxisome biogenesis (Marelli *et al.* 2004; Akşit and van der Klei 2018) where Pex25 overexpression has been shown to drive formation of juxtaposed elongated peroxisomes (Rottensteiner *et al.* 2003; Tam *et al.* 2003; Huber *et al.* 2012). The tubules are similar to those in *Ogataea* *polymorpha* lacking Dnm1, suggesting Pex25-GFP overexpression may also block fission machinery (Smith *et al.* 2002; Nagotu *et al.* 2008; Akşit and van der Klei 2018). Together, these results highlight the rich information obtainable from overexpression experiments facilitated by this toolkit.

This toolkit joins the array of methods available for overexpression studies. Plasmid libraries exist that contain fragmented genomic DNA systematically expressed on high-copy 2µ vectors (Rose and Broach 1990), genes expressed from both low-copy vectors (Ho *et al*. 2009) and 2µ vectors (Magtanong *et al*. 2011) and genes under the control of the galactose-inducible *GAL1* promoter (Gelperin *et al*. 2005). While largely covering the genome and being easily transformed, these approaches suffer from copy number variation and instability. More recently, integrated genome-scale libraries have been made with either β-estradiol-inducible promoters (Arita *et al.* 2021) or easily swappable 5′ or 3′ insertions at each gene that can be used for tagging and introducing strong or inducible promoters (Yofe *et al*. 2016). While these 2 systems are particularly well suited for genome-wide overexpression studies, they cannot easily be applied in a new strain background. In contrast, our system can stably express any gene, including nonyeast genes, either tagged or untagged at multiple defined expression levels and copy numbers in any target background with little more effort than introducing a plasmid. Thus while, ill-suited for genome-wide screens, our approach should be valuable in many situations, particularly when a metabolic engineer is seeking to tune expression levels.

Though the MyLO toolkit provides a strong foundation for basic CRISPR-Cas9 genome editing in yeast, making stable genomic integrations almost as easy as introducing a plasmid, it could be expanded in numerous ways. These expansions could include adding new integration cassettes with multipurpose homology arms for different sets of sites, additional promoters including those allowing regulation of gene expression, and the inclusion of alternative compartmental markers. Furthermore, inverting the order of homology regions on one of the arms should allow a single cassette to repair multiple sites enabling single-step multicopy introduction of a cassette, as a cassette integrated at a single location could have the left and right homology arms required to repair cut sites introduced by Cas9 at other locations. The pCAS vectors could also be modified to include new smaller or higher fidelity CAS proteins and should be easily adaptable to the method presented by Zhang *et al.* for multiplexing of gRNAs (Kim *et al.* 2017; Casini *et al.* 2018; Zhang *et al.* 2019; Xu *et al.* 2021). Indeed, the increased efficiency and simplicity of the split selection pCAS strategy could further improve current CRISPR multiplexing capabilities.

## Data availability

Toolkit plasmids (pBBK01-96) are available from Addgene, while other strains and plasmids are available on request. The authors affirm that all data necessary for confirming the conclusions of the article are present within the article, figures, and tables.

Supplemental material is available at *G3* online.

## Supplementary Material

jkac154_Supplemental_Material_Legends

jkac154_Supplemental_Figure_S1

jkac154_Supplemental_Table_S1

jkac154_Supplemental_Table_S2

jkac154_Supplemental_Table_S3

jkac154_Supplemental_File_S1

jkac154_Supplemental_File_S2
