# Supplementary material for: The MyLO CRISPR-Cas9 toolkit: a markerless yeast localization and overexpression CRISPR-Cas9 toolkit
Source: G3 (Bethesda). 2022 Jun 16;12(8):jkac154. doi: 10.1093/g3journal/jkac154 (PMC9339301; doi:10.1093/g3journal/jkac154)
Supplement: jkac154_Supplemental_Material_Legends [file jkac154_supplemental_material_legends.docx]

**SUPPLEMENTARY DOCUMENTS**

**FIGURE S1**. **Additional measures of fluorescence.**

**TABLE S1 – MyLO Toolkit Plasmids**

**TABLE S2 – Primers used**

**TABLE S3 – Yeast strains used**

**FILE S1. Plasmid construction details**

**FILE S2. MyLo CRISPR Toolkit instructions and recommended protocols**
