# Supplementary material for: The MyLO CRISPR-Cas9 toolkit: a markerless yeast localization and overexpression CRISPR-Cas9 toolkit
Source: G3 (Bethesda). 2022 Jun 16;12(8):jkac154. doi: 10.1093/g3journal/jkac154 (PMC9339301; doi:10.1093/g3journal/jkac154)
Supplement: jkac154_Supplemental_Figure_S1 [file jkac154_supplemental_figure_s1.pdf]

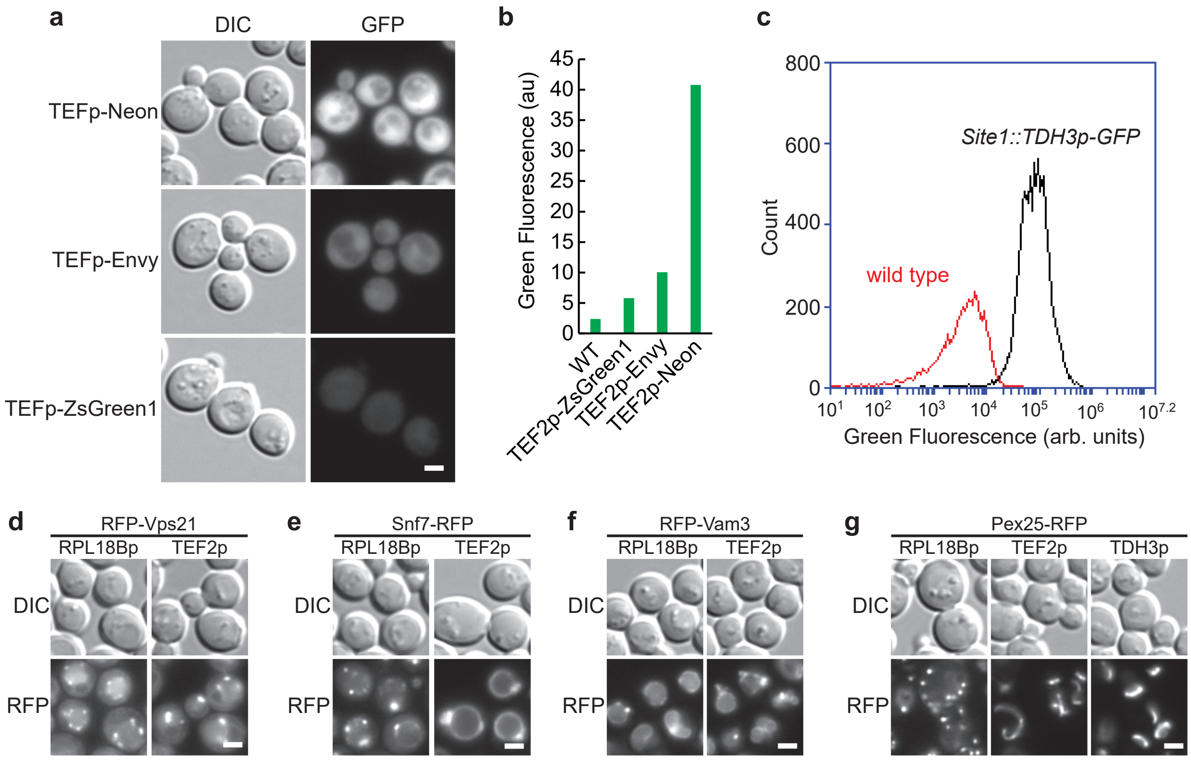

FIGURE S1. Additional measures of fluorescence. The relative green fluorescence of three tested GFPs expressed from the intermediate *TEF2p* promoter was assessed by **(a)** microscopy and **(b)** flow cytometry. n = 1; 10,000 cells/strain. **(c)** Representative histograms of green fluorescence intensity measured by flow cytometry of wild type cells and a strain with *TDH3p-GFP* integrated at Site 1. These data correspond with a subset of Figure 2f Fluorescence microscopy of yeast with RFP-tagged markers **(d)** RFP-Vps1, **(e)** Snf7-RFP, **(f)** RFP-Vam3 and **(g)** Pex25-GFP displaying changes in localization as promoter strength increases *RPL18Bp* < *TEF2p* < *TDH3p*. n = 2. Scale bars indicate 2 μm.
